# Effect of the cholinergic system of the lateral periaqueductal gray (lPAG) on blood pressure and heart rate in normal and hydralazine hypotensive rats

**DOI:** 10.22038/IJBMS.2023.66838.14660

**Published:** 2023

**Authors:** Atiyeh Ghorbani, Reza Mohebbati, Alireza Rahimi, Vida Alikhani, Mohammad Naser Shafei

**Affiliations:** 1 Department of Physiology, Faculty of Medicine, Mashhad University of Medical Sciences, Mashhad, Iran; 2 Applied Biomedical Research Center, Mashhad University of Medical Sciences, Mashhad, Iran; 3 Material Science and Metallurgy Engineering, Islamic Azad University-Karaj Branch; 4 Division of Neurocognitive Sciences, Psychiatry, and Behavioral Sciences Research Center, Mashhad University of Medical Sciences, Mashhad, Iran

**Keywords:** Acetylcholine, Blood pressure, Heart rate, Heart rate variability, Hydralazine, Lateral periaqueductal gray

## Abstract

**Objective(s):**

Due to the presence of the cholinergic system in the lateral periaqueductal gray (lPAG) column, the cardiovascular effects of acetylcholine (ACH) and its receptors in normotensive and hydralazine (HYD) hypotensive rats in this area were evaluated.

**Materials and Methods:**

After anesthesia, the femoral artery was cannulated and systolic blood pressure (SBP), mean arterial pressure (MAP), heart rate (HR), and also electrocardiogram for evaluation of low frequency (LF) and high frequency (HF) bands, important components of heart rate variability (HRV), were recorded. ACH, atropine (Atr, a muscarinic antagonist), and hexamethonium (Hex, an antagonist nicotinic) alone and together microinjected into lPAG, changes (Δ) of cardiovascular responses and normalized (n) LF, HF, and LF/HF ratio were analyzed.

**Results:**

In normotensive rats, ACH decreased SBP and MAP, and enhanced HR while Atr and Hex did had no effects. In co-injection of Atr and Hex with ACH, only ACH+Atr significantly attenuated parameters. In HYD hypotension, ACH had no affect but Atr and Hex significantly improved the hypotensive effect. Co-injection of Atr and Hex with ACH decreased the hypotensive effect but the effect of Atr+ACH was higher. In normotensive rats, ACH decreased nLF, nHF, and nLF/nHF ratio. These parameters in the Atr +ACH group were significantly higher than in ACH group. In HYD hypotension nLF and nLF/nHF ratio increased which was attenuated by ACH. Also, Atr+ACH decreased nLF and nLF/nHF ratio and increased nHF.

**Conclusion:**

The cholinergic system of lPAG mainly via muscarinic receptors has an inhibitory effect on the cardiovascular system. Based on HRV assessment, peripheral cardiovascular effects are mostly mediated by the parasympathetic system.

## Introduction

The periaqueductal gray (PAG) is a midbrain region involved in various functions including regulation of autonomic response, defense and emotional responses, as well as learning and modulation of pain (1-3). Based on anatomical and physiological functions, the PAG region is divided into four longitudinal columns of neurons, namely dorsomedial PAG (dmPAG), dorsolateral PAG (dlPAG), lateral PAG (lPAG), and ventrolateral PAG (vlPAG) (3). It has been shown that each one of the PAG columns has different functions in the control of the cardiovascular system. For example, it has been revealed that dlPAG and vlPAG have excitatory and inhibitory effects on the cardiovascular system, respectively {Dampney, 2016 #3}{Dampney, 2015 #8}.

One of the columns whose cardiovascular effects are less known is lPAG. Primary studies have shown this column is associated with defense reaction, and its activation causes hypertension and tachycardia {Dampney, 2015 #8}. lPAG receives afferents from both superficial and deep laminae of the dorsal horn (4), and via the nucleus para-retroambiguus projects to the intermediolateral cell column (IML) of the spinal cord (5). LPAG also has connections to the rostral ventrolateral medulla (RVLM), an essential area for cardiovascular regulation (3), NTS, and vagal preganglionic neurons. In addition, it has been revealed that lateral/dorsolateral PAG neurons are major components of the central pathways that mediate cardiovascular responses stimulated by the activation of the dorsomedial hypothalamus (DMH) nucleus (6). 

Acetylcholine (ACH) is a neurotransmitter whose presence in lPAG (7) has been indicated. According to previous studies, ACH plays an important role in regulating cardiovascular function within the nervous system (8). The microinjection of ACH into the RVLM area could increase blood pressure as well as heart rate (HR)(9). In the case of normotensive rats, its microinjection into the cuneiform nucleus (CnF) or pedunculopontine tegmental nucleus (PPT) has decreased cardiovascular parameters (10). ACH is also involved in cardiovascular regulation under hypotension conditions. For example, the cholinergic system of the posterior hypothalamic nucleus (PHN) shows an improvement in the cardiovascular response induced by hemorrhage.

Hypotension is a low blood pressure condition that can deprive the brain and other vital organs of oxygen and nutrients, leading to a life-threatening condition. Hypotension evokes the release of numerous neurotransmitters in brain areas that are involved in cardiovascular regulation such as RVLM, NTS PVN, and vlPAG. To evaluate the role of central areas during hypotension, several methods were used. One well-known method is hypotension induced by Hydralazine (HYD) (11, 12), which by direct relaxation of the smooth muscle in the arterial vessels induces hypotension (12). HYD also reduces blood pressure, leukocyte migration, apoptosis, and damage to heart tissue (13, 14).

Heart rate variability (HRV) is a non-invasive method to evaluate the function of cardiovascular control by the autonomic nervous system (ANS) in various conditions. Hence, the analysis of HRV is beneficial to the diagnosis of cardiovascular diseases and helpful in preventative medicine and sports therapy. The spectral HRV parameters achieved from the R-R interval data of electrocardiograms (ECG) are of great importance to be statistically calculated and analyzed. The power spectra of the R-R intervals have two important low (LF; 0.20 to 0.75 Hz) and high (HF; 0.75 to 2.50 Hz) heart rate frequencies. HF indicates the neural activity of the parasympathetic system while LF shows both sympathetic and parasympathetic effects on the heart function. Moreover, the ratio of LF to HF (LF/HF) is an important factor in the regulation of the heart function that shows the balance between sympathetic and parasympathetic systems (14-16).

Due to the presence of cholinergic receptors in the lPAG (17, 18) and also the involvement of this receptor in cardiovascular regulation (19, 20), we investigated the possible role of the cholinergic system of lPAG on cardiovascular activity as well as HRV under normotensive and hypotension induced by HYD in anesthetized rats. 

## Materials and Methods

***Animals and groups***

In this experimental study, 60 male Wistar rats (250±20 g) were used. The animals were kept in standard conditions (12-hr light/dark cycle with complete access to food and drinking water). All procedures in this experiment were approved by the ethical research committee of Mashhad University of Medical Sciences (IR.MUMS.MEDICAL.REC.1400.403). 

***Drugs and animal groups***

The drugs used in this experiment were urethane (an anesthetic drug, Merck, USA), acetylcholine hydrochloride (ACH, an agonist of cholinergic system, Sigma Aldrich Chemical Co., USA), atropine (Atr, an antagonist of muscarinic receptors), hexamethonium (Hex, an antagonist of nicotinic receptor(21), and hydralazine (HYD), a peripheral arterial vasodilator that induces hypotension) (22).

The rats were randomly assigned to (A) normotensive and (B) hypotensive groups (n=6 for each group). 

(A) Normotensive groups: 

1. Control group: Saline microinjected into lPAG

2. ACH group: ACH (150 nmol) microinjected into lPAG

3. Atr group: Atr (9 nmol) microinjected into lPAG 

4. Hex group: Hex (300 nmol) microinjected into lPAG 

5. ACH+Hex group: First, Hex was microinjected and after 2 min, ACH was microinjected into lPAG

6. ACH+Atr group: First, Atr was microinjected, after 2 min, ACH was microinjected into lPAG 

(B) Hypotensive groups:

1. HYD group: HYD (10 mg/kg) injected intravenously (IV)(23) 

2. HYD+ACH group: First, HYD (IV) was injected and after 2 min, ACH was microinjected into lPAG 

3. HYD+Atr group: First, HYD was injected (IV) and after 2 min, Atr was microinjected into lPAG 

4. HYD+Hex group: First, HYD was injected (IV), and then ACH was microinjected into lPAG 

All drugs were dissolved in saline and those doses were selected based on previous studies(24). The volume for intravenous injections was 0.5 ml and 100–150 nl for microinjection into lPAG (21)

***Cardiovascular parameter measurement***

Cardiovascular responses were recorded according to the methods described in previous studies (25). Briefly, after administering anesthesia with urethane (1.5 g/kg, IP) (26), the left femoral artery was cannulated by a blue angiocatheter (22-gauge, Indian Co) filled with heparinized saline (50 u/ml) (27). Then the angiocatheter was connected to a blood pressure transducer, and systolic blood pressure (SBP), mean arterial pressure (MAP), and HR were continuously recorded by a power lab system (ID instrument, Australia) (28). For intravascular (IV) injection of HYD (10 mg/kg), the right femoral vein was cannulated (29). 

***Heart rate variability analysis***

For HRV analysis, the data of standard II lead of ECG was recorded by a Power Lab system (4/25T, AD Instruments, Bella Vista, NSW, Australia) and HRV was measured in the frequency domains including the LF power at 0.20 to 0.75 Hz, the HF power at 0.75 to 2.50 Hz, and the total power (TP) at 0 to 3.00 Hz (15). To better analyze the powers, we calculated the nLF and the nHF values. The nLF is an index of sympathetic function and nHF is an index of parasympathetic function, which are calculated by the following equations: nLF=100×LF/(TP−VLF); nHF=100×HF/ (TP−VLF). These values are expressed as normalized units (nu)(15, 30). The nLF/nHF ratio shows the sympathetic-parasympathetic balance.

***Drug microinjection***

For the microinjection of drugs, the animals were placed in a stereotaxic apparatus (Stoelting, USA) and a hole was drilled in their skulls above lPAG based on the coordination of this column in the Paxinos atlas (AP: -7.56 mm, L: +1 mm, H: -5.4 mm ventral from the skull surface)(31) 

***Data analysis***

In all groups, comparisons between maximal changes (sampled before and after) evoked in blood pressure and HR during the injection of drugs were calculated and expressed as mean±SEM. One-way analysis of variance (ANOVA) followed by Tukey’s *post hoc* test was used for statistical analysis. Analyses were performed by Instant-Quantified Self 6.1.8. The level of significance was taken as *P*<0.0*5*. 

## Results

***Effects of saline microinjection into lPAG on the cardiovascular responses*** ***in normotensive rats***

First, SBP, MAP, and HR were recorded and then saline was microinjected into lPAG and the changes in cardiovascular parameters were evaluated. The results indicated that saline did not significantly alter SBP, MAP, and HR compared with pre-injection.

***Effects of Atr and ACH+Atr microinjection into lPAG on cardiovascular responses in normotensive rats***

**Figure 1:**
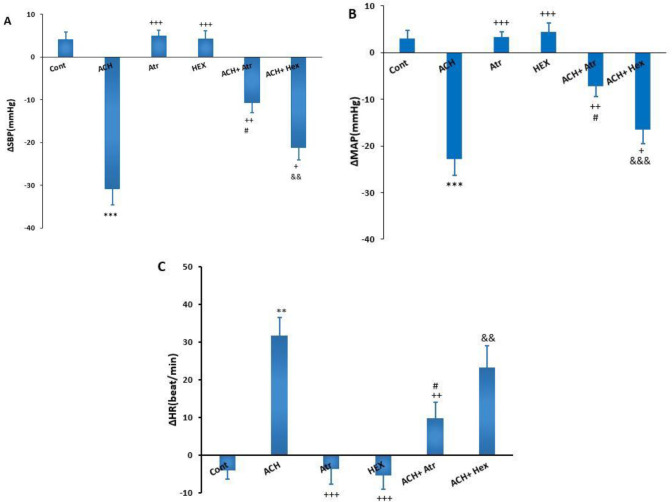
Changes (∆) of cardiovascular responses induced by ACH, Atr, and Hex, Hex+ACH, and Atr+ACH microinjected into lPAG in normotensive rats. (a) ΔSBP, (b) ΔMAP, and (c) ΔHR. One-way ANOVA followed by Tukey’s post hoc test; n= 6. *** *P<*0.001 vs control; ^+^*P<*0.05, ^++^*P<*0.01, ^+++ ^*P<*0.001 vs ACH; ^#^*P<*0.05 Atr vs ACH+ Atr; ^&& ^*P<*0.01 and ^&&&^ *P<*0.001 Hex vs ACH+ Hex

SBP: Systolic Blood Pressure; HR: Heart Rate; MAP: Mean Arterial Pressure; ACH: Acetylcholine; Atr: Atropine; Hex: Hexamethonium

After the stabilization of the cardiovascular parameters, ACH, Atr, and Atr+ACH were microinjected into lPAG in separate groups. The microinjection of ACH significantly decreased blood pressure (ΔSBP: -30.83±3.7 mmHg vs saline: 3.4±1.7 mmHg; ΔMAP: -22.66±3.7 mmHg vs saline: 2.99±1.7 mmHg; *P*<0.001) and increased ΔHR (31.67±6.9 beat/min vs saline: -3.97±2.3 beat/min; *P*<0.01, Figure 1). The microinjection of Atr alone into lPAG did not significantly alter the cardiovascular parameters (ΔSBP: 5±1.3 mmHg, ΔMAP: 3.2±1.6 mmHg, and ΔHR: -3.7±4 beat/min) compared with the control group. The microinjection of Atr before ACH (ACH+Atr group) into lPAG significantly attenuated the effect of ACH on ΔSBP (ACH+Atr: -10.6±2.4 mmHg vs ACH: -30.83±3.7 mmHg; *P*<0.01) and MAP (ACH+Atr: -7.1±1.98 mmHg vs ACH: -22.66±3.7 mmHg; *P*<0.01). ΔHR was also significantly decreased by Atr (ACH+Atr: 9.8±4.28 beat/min vs ACH: 31.67±6.9 beat/min; *P*<0.01, Figure 1). The cardiovascular responses in the Atr+ACH group also were more significant than those in the Atr group (*P*<0.05).

***Effects of Hex and ACH + Hex microinjection into lPAG on cardiovascular responses in normotensive rats***

In this experiment, Hex alone and with ACH (ACH+Hex group) were microinjected into lPAG separately. Hex alone did not significantly affect ΔSBP and ΔMAP compared with the control group (ΔSBP: 4.16±2 mmHg vs saline: 3.4±1.7 mmHg, ΔMAP: 4.16±2 mmHg vs saline: 2.99±1.7 mmHg, and ΔHR: -5.33±3.2 beat/min vs saline: -3.97±2.3 beat/min, n=6). However, the cardiovascular effects of Hex were more significant compared with the ACH group (*P*<0.001). The microinjection of Hex before ACH (Hex+ACH group) significantly attenuated the hypotensive effect of ACH (ΔSBP: Hex+ACH: -21.16±2.95 mmHg vs ACH: -30.83±3.7 mmHg, *P*<0.05 and ΔMAP: Hex+ACH: -21.16±2.95 mmHg vs ACH: -22.66±3.7 mmHg, *P*<0.05). The change of ΔHR in the ACH+Hex group was also not significant compared with the ACH group (Hex+ACH: 23.16±5.8 beat/min vs ACH: 31.67±6.9 beat/min, *P*>0.05, Figure 1).

***Effect of intravenous injection of HYD on the cardiovascular responses***

**Figure 2:**
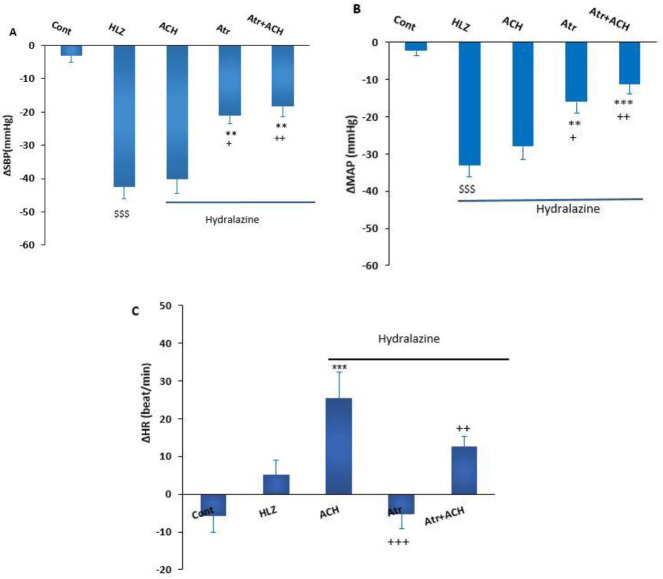
Cardiovascular responses induced by microinjection of ACH, Atr, and ACH+Atr into lPAG in hydralazine (HYD) hypotensive rats. One-way ANOVA followed by Tukey’s post hoc test; n= 6. ** *P<*0.01 *** *P<*0.001 compared with HYD; ^+^*P<*0.05; ^++^*P<*0.01; ^+++^*P<*0.01, compared with ACH; ^$$$^ *P<*0.001 compared with Control

To induce hypotension, HYD (10 mg/kg) was injected intravenously (IV). The record of cardiovascular responses after HYD injection indicated that ΔSBP (HYD: -42.4±3.7 mmHg *vs* Saline: 3.4±1.7 mmHg) and ΔMAP (HYD: -33±3.2 mmHg *vs* saline: 2.99±1.7 mmHg, *P*<0.0*1*) significantly decreased compared with saline. ΔHR also decreased but this effect was not significant compared with the saline group (HYD: -12.4±6.2 beat/min *vs *saline: -3.97±2.3, Figure 2). 

***Cardiovascular responses after ACH, Atr, and Hex microinjection into lPAG in HYD hypotensive rats***

In this experiment, the cardiovascular effect of ACH, Atr, and Atr+ACH in hypotension induced by HYD was examined. The microinjection of ACH into lPAG in the presence of HYD did not significantly alter ΔSBP (ACH+HYD: -40.4±4.52 mmHg *vs* HYD: -42.4±3.7 mmHg) and ΔMAP (ACH+HYD: -28.3±3.6 mmHg *vs* HYD: -33±3.2 mmHg). ΔHR in this group significantly increased compared with the HYD group (ACH+HYD: 31.8±7 beat/min *vs* HYD: -12.45±46.2 beat/min, *P*<0.001). Microinjection of Atr into lPAG significantly improved the hypotension induced by HYD (ΔSBP (Atr+HYD: - 21.316.6±3.6 mmHg *vs* HYD: -42.4±3.7 mmHg, *P*<0.0*1*)) and ΔMAP (Atr+HYD: -16.6±2.4 mmHg *vs* HYD: -33±3.2 mmHg, *P*<0.0*1*). The changes of ΔSBP and ΔMAP in the Atr+HYD group were also significantly lower than in the ACH+HYD group (*P*<0.05). ΔHR in this group significantly decreased compared with the HYD group (Atr+HYD: -5.2±4 beat/min *vs* HYD: 5±4 beat/min, *P*<0.05, Figure 2). 

Co-injection of ACH+Atr after hypotension significantly improved the decreased ΔSBP (HYD+Atr+ACH: -18.25± -3.16 mmHg *vs* HYD: -42.4±3.7 mmHg) and ΔMAP (HYD+Atr+ACH: -11.25±2.7 mmHg *vs* HYD: -33±3.2 mmHg). Moreover, the effect of ΔHR significantly increased compared with HYD (HYD+Atr+ACH: 10.5±2.8 beat/min *vs* HYD: -12.45±46.2 beat/min, *P*<0.05). Comparison of the changes between ΔMAP and ΔSBP in the HYD+Atr+ACH group and the HYD group showed that these parameters significantly reduced compared with the ACH+HYD group (*P*<0.0*1*). In addition, the increase of ΔHR in the HYD+ACH group was significantly attenuated by the co-injection of Atr+ACH (Atr+ACH+HYD group, *P*<0.01, Figure 2).

**Figure 3:**
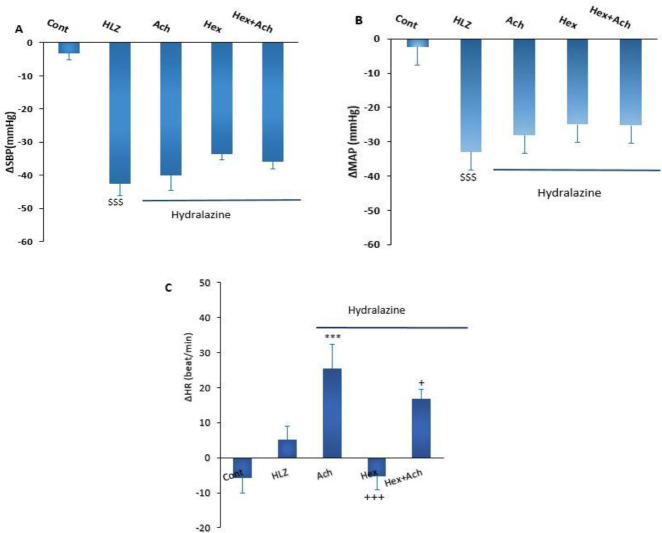
Cardiovascular responses induced by microinjection of ACH, Hex, and ACH+Hex into lPAG in hydralazine hypotensive rats. One-way ANOVA followed by Tukey’s *post hoc* test; (n=5-7). *** *P<*0.001 compare to HYD; ^+^*P<*0.05 and ^+++^*P<*0.001 Compared with ACH; ^$$$^ *P<*0.001 compared with Control

The microinjection of Hex into lPAG significantly improved the decrease of ΔSBP (Hex+HYD: -26.19±2 mmHg *vs* HYD: -42.4±3.7 mmHg*, P<*0.01*)) and ΔMAP (Hex+HYD: -16.6±2.4 mmHg vs HYD: -33±3.2 mmHg, P<*0.01*)) induced* by HYD. The decreased ΔSBP and ΔMAP in the ACH+HYD group also did not significantly change after the co-injection of ACH+Hex (*P*>0.05). In addition, the increase of ΔHR in the HYD+ACH group was attenuated by the co-injection of Hex+ACH (Hex+ACH+HYD group), but this effect was not significant (Figure 3).

***HRV in normotensive rats***

**Figure 4:**
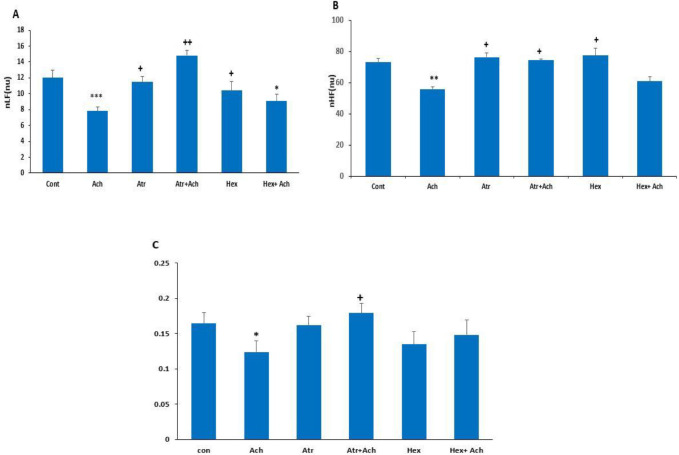
Heart rate variability (HRV) analysis after microinjection of ACH, Atr, Hex, Atr+ACH, and Hex+ACH into lPAG in normotensive rats. Normalized low frequency (nLF, A), normalized high frequency (nHF, B), and nLF/nHF ratio (nLF/nHF, C). Data are the mean±SEM (n=6). * *P<*0.05; ** *P<*0.01; *** *P<*0.001 vs control; ^+ ^*P<*0.05; ^++^ *P<*0.01 vs ACH

In this experiment, spectral bands of HRV (nLF and nHF) and their ratio (nLF/nHF) after the microinjection of saline, ACH, Atr, Atr+ACH, Hex, and Hex+ACH into lPAG were examined. The microinjection of ACH significantly decreased nLF compared with the saline group (*P*<0.001) and the co-injection of Atr+ACH significantly increased nLF compared with ACH (*P*<0.0*1*) and control (*P*<0.05) groups. In the HEX+ACH group, nLF was significantly decreased compared with the control group (*P*<0.05). In the ACH group, nHF significantly decreased compared with control (*P*<0.0*1*) and in the Atr+ACH and Hex+ACH groups significantly increased compared with the ACH group (*P*<0.05). The nLF/nHF ratio in the ACH group was significantly lower than in the control group (*P*<0.05). Furthermore, this ratio in the Atr+ACH group was significantly higher than in the ACH group (*P*<0.05, Figure 4). 

***HRV in hydralazine hypotensive rats***

**Figure 5:**
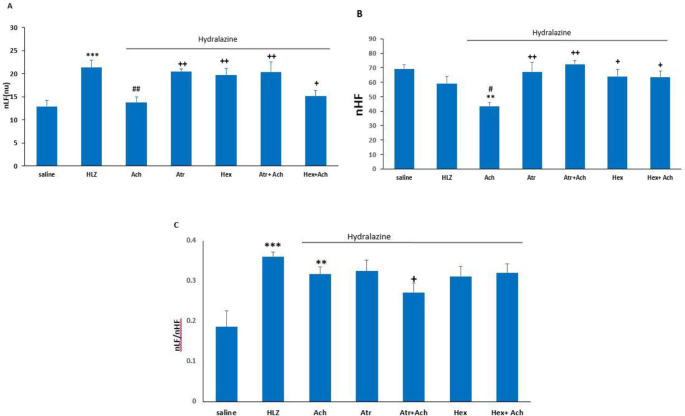
Heart rate variability (HRV) analysis after microinjection of ACH, Atr, Hex, Atr+ACH, and Hex+ACH into lPAG in hydralazine hypotensive rats. Normalized low frequency (nLF), normalized high frequency (nHF), and nLF/nHF ratio (nLF/nHF). Data are mean±SEM (n=6). * *P<*0.05; ** *P<*0.01; *** *P<*0.001 vs control; ^+^ *P<*0.05; ^++^ *P<*0.01 vs ACH; ^#^ *P<*0.05; ^##^ *P<*0.01 vs HYD

In this part of the study, hypotension was first induced by HYD and after that, spectral bands of HRV (nLF and nHF) and their ratio (nLF/nHF) were analyzed following the microinjection of ACH, Atr, Atr+ACH, Hex, and Hex+ACH into lPAG. Figure 5 indicates the HRV analysis in HYD hypotensive rats. As can be observed, the nLF band and the nLF/nHF ratio in the HYD group were significantly higher than in the control group (*P*<0.001), but the nHF band was not significantly decreased. In the presence of HYD, the nLF and nHF bands in the ACH group were significantly decreased compared with the HYD group (*P*<0.0*1*) while the nLF/nHF ratio did not significantly change. Moreover, in the Atr and Hexa groups alone as well as the HEX+ACH group, nLF did not significantly change compared with the HYD group, but it was significantly different from the control and ACH groups (*P*<0.01). nHF in these groups was significantly changed compared with the ACH group but was not significantly different from the control and HYD groups. In the Atr+ACH group, nLF and the nLF/nHF ratio significantly decreased (*P*<0.01 and *P*<0.05, respectively) and nHF increased (*P*<0.05) compared with the HYD group. In this group, nLF was not significantly changed, but nHF was significantly different (*P*<0.0*1*) compared with the ACH group (Figure 5). 

## Discussion

In this study, we discussed the cardiovascular effect of the cholinergic system of the lPAG column. Our results indicated that in normotensive rats, the microinjection of ACH decreased SBP and MAP while increasing HR. In addition, the microinjection of ACH into lPAG made no change to hypotension induced by HYD but significantly enhanced HR. We also observed that the cardiovascular response triggered by direct cholinergic activation of lPAG neurons is mostly mediated by muscarinic receptors. Assessment of HRV also indicated that both nLF and nHF significantly reduced in normotensive and hypotension rats and atropine improved these effects. 

LPAG is known to contribute to the defense reaction (32). This contribution is concerned with either active (visceral vasoconstriction, freezing, flight, and increased cardiovascular responses) or passive (immobility and sympathoinhibitory responses) coping strategies. It has been reported that lPAG is more associated with active strategies which are adaptive for coping with threatening situations via elevated cardiovascular and respiratory responses (3, 33). Consistent with this finding, microinjection of D, L-homocysteic acid (DLH) into lPAG has led to tachypnea and inspiratory apneusis (34). In this experiment, we evaluate the cardiovascular effect of ACH in lPAG. The present results show that microinjection of ACH into lPAG decreased blood pressure and increased HR in normotensive rats. In line with our results, a previous study also reported the depressor and bradycardia effects of ACH microinjection into lPAG (17). The fact that the injection of ACH into lPAG column reduces cardiovascular effects, suggests an inhibitory cardiovascular effect of lPAG cholinergic system. The mechanism of this effect of the cholinergic system of lPAG is currently unknown but can be considered by the mediation of neurons or projections of areas involved in cardiovascular function. Previous studies revealed that ACH has an inhibitory effect on the cardiovascular system (19, 26). LPAG has several neurons, some of which are inhibitory such as GABAergic interneurons in the nervous system (2, 35). Therefore, activation of these inhibitory neurons enables ACH to decrease blood pressure. Also, ACH comes across as being able to interact with other neurotransmitters such as nitric oxide and glutamate (36). This assumption nevertheless needs further investigation to be confirmed. LPAG area receives afferents from the spinal cord and the spinal trigeminal nucleus and directly projects to the RVLM, an important area in regulating cardiovascular function (37, 38). Thus, it is hypothesized that the projection of lateral PAG to the RVLM is inhibited by the cholinergic system, otherwise, there is an inhibitory projection from this area to the RVLM region. On the other hand, HR was increased by the microinjection of ACH into lPAG area. This effect of ACH might be mediated by the baroreflex activity. It could also be attributed to the fact that HR regulation is independent of blood pressure and mediated by its projection to the vagal preganglionic neurons and the nucleus of the solitary tract (NTS)(39). The PAG receives input from the dorsomedial hypothalamus (DMH)(3, 32). In another study, activation of neurons in the DMH nucleus by microinjection of GABA_A_ receptor antagonist, bicuculline methiodide (BMI), or excitatory amino acids resulted in cardiovascular and behavioral responses, resembling those observed after activation of l/dlPAG (6, 40, 41). It was suggested that neurons in l/dlPAG constitute responsible downstream effectors for cardiovascular changes elicited by the DMH. Accordingly, it can be suggested that the projection of lPAG to DMH is inhibited by the cholinergic system.

Relation of PAG with raphe pallidus (RPa) has been reported (42). In research conducted by Moraes *et al*., the cardiac output increases during defensive behaviors was ascribed to the PAG-RPa pathway (43). Therefore, involvement of the PAG-RPa pathway in HR elevation in the cholinergic stimulation of lPAG sounds plausible, which merits further investigation.

In another experiment, Atr (an antagonist of muscarinic receptors) and Hex (an antagonist of nicotinic receptors) were separately microinjected into lPAG to determine the effect of ACH receptor (nicotinic or muscarinic) on the cardiovascular function. In normotensive rats, the microinjection of Atr or Hex into lPAG *per se* did not significantly affect HR and blood pressure. The low secretion of ACH during anesthesia may be the reason behind this lack of cardiovascular intervention. To confirm the individual role of Atr and Hex receptors, ACH was co-injected with each separately. The results showed that Atr was fairly effective in improving the decreased SBP and MAP by ACH. By contrast, Hex had no significant effect on the cardiovascular changes induced by ACH. Thus, the cardiovascular intervention of ACH injection into lPAG was mostly mediated by muscarinic receptors. These results are consistent with those of previous studies reporting that the muscarinic receptor coordinates cardiovascular activity as the main receptor in the brain (10, 26, 44). It was reported that the cardiovascular effect of the cholinergic system in the CnF nucleus was mediated by muscarinic receptors (10).

Due to lPAG involvement in physical stresses, another experiment was designed to examine the role of lPAG cholinergic system in the HYD hypotensive model of hypotension. HYD is a direct arteriole vasodilator that manages hypotension by blocking the inositol trisphosphate (IP3)-dependent release of calcium from smooth muscle sarcoplasmic reticulum and inhibiting arterial smooth muscle contraction by myosin phosphorylation (15, 45). The intravenous injection of HYD (10 mg/kg) resulted in a drop in blood pressure (about 40 mmHg) and no considerable change in HR.

Similarly, injection of ACH into lPAG did not significantly influence HYD-induced hypotension. Based on our results, HYD had a similar hypotensive influence to that of ACH. To achieve further results, Atr (a muscarinic antagonist) was twice injected into lPAG, with and without ACH, after HYD hypotension. It was indicated that hypotension was improved in the presence of Atr alone. Therefore, we concluded that the secretion of ACH is elicited during hypotension when its hypotensive effect is mediated by muscarinic receptors. In addition, analogous to the single injection of Atr, the co-injection of ACH+Atr into lPAG attenuated the hypotensive responses to HYD. In comparison, Atr was observed to have a higher effect in the hypotensive condition than in the normotensive condition. The most plausible explanation of this observation is that there is a low release of ACH in normal condition but it becomes elevated during hypotension and thus contributes to it. This is while the effect of Atr injection differs under hypotension conditions. 

Moreover, the single injection of Hex and the co-injection of Hex+ACH into lPAG did not affect the hypotension induced by HYD. This indicates that the nicotinic receptors do not intervene in cardiovascular responses during hypotension in this area. 

In this experiment microinjection of ACH into lPAG also made a rise in HR, possibly as a baroreflex response due to the depressor effect of ACH. Although ACH microinjection did not make a change in blood pressure, it significantly raised HR in HYD-induced hypotension. These results revealed that the mechanism of HR regulation by the cholinergic system is different from that of blood pressure regulation. It is claimed that PAG has projections to the dorsal motor nucleus of the vagus (DMV) and NTS. Therefore, it is suggested to investigate the projection of lPAG to these areas in relation to HR regulation for future studies.

In another experiment, we evaluated HRV changes due to the microinjection of ACH and found decreased values of normalized LF, HF, and LF/HF ratio. In anesthetized rats, HR oscillations in the LF band were indicative of both sympathetic and parasympathetic modulations, whereas HR oscillations in the HF band were indicative of only parasympathetic modulation. HRV, especially the LF and HF components, was reduced after microinjection of ACH. Interestingly, in ACH rats, the already decreased LF band of HRV was further reduced merely by the parasympathetic blockade. 

In evaluating HRV under hypotension conditions, HYD enhanced normalized values of LF and LF/HF and diminished HF. While the attenuated parasympathetic activity corresponded to the reduced amount of HF, the augmented sympathetic activity was associated with enhanced value of LF and increased ratio of LF/HF (46). A cross-spectral analysis was done on HR and arterial pressure to evaluate the baroreflex function which proved to be correlated with the sympathetic influence on HRV in the LF band (47, 48). The bradycardic effect found in this study could be exclusively attributed to hypertension-induced baroreflex activation. It was nevertheless expected that resetting mechanisms would allow HR to return to normal within a few days, as demonstrated in other experimental models of hypertension (49). All the above-mentioned factors somehow contribute to a higher risk of cardiovascular diseases, especially heart arrhythmia (50).

Compared with the HYD group, the ACH+HYD group had a lower LF as well as HF, increased sympathetic activation in heart failure, but a decreased LF variability and a decreased LF/HF ratio. In ACH hypotensive rats, a remarkable tachycardia was promoted by the increase in sympathetic activity, conflicting with the decrease in LF variability in HR as well as LF/HF ratio. This conflict was formerly observed in instances of sharply increased sympathetic activity such as heavy physical activity (51) or severe cardiac failure (52). In such a situation, all homeostatic mechanisms of the circulatory system are outfitted at close to the maximum with little reserve to maintain cardiovascular variability. In this model of hypertension, the cause of reduced HRV at the LF band remained unknown. However, in an alternative model (52), reduced LF band in HRV was assigned to arterial baroreflex dysregulation, central autonomic modulation abnormality, or neurotransmitter sensitivity alteration in the target organ.

Atr: atropine; Hex: Hexamethonium; ACH: Acetylcholine; Cont: control

Atr: atropine; Hex: Hexamethonium; ACH: Acetylcholine; HYD: hydralazine

## Conclusion

The present results demonstrated an inhibitory impact of lPAG cholinergic system on the cardiovascular system, mostly mediated by muscarinic receptors. Additionally, in spite of the low release of Ach in normotensive function, its release from lPAG could be evoked by hypotension. Furthermore, our study on the effect of cholinergic mediation resulted in reduced LF and HF in normotensive and hypotensive cases, likely suggestive of parasympathetic activation.

## Authors’ Contributions

G A performed methodology, investigation, and formal analysis. G A, M R, and R R wrote and prepared the manuscript.S MN conceived the study and supervised.

## Conflicts of Interest

The authors declare that they have no conflicts of interest.
